# Integrating multi-omics data to reveal the effect of genetic variant rs6430538 on Alzheimer's disease risk

**DOI:** 10.3389/fnins.2024.1277187

**Published:** 2024-03-18

**Authors:** Shizheng Qiu, Meili Sun, Yanwei Xu, Yang Hu

**Affiliations:** ^1^School of Computer Science and Technology, Harbin Institute of Technology, Harbin, China; ^2^Beidahuang Industry Group General Hospital, Harbin, China; ^3^Beidahuang Group Neuropsychiatric Hospital, Jiamusi, China

**Keywords:** *TMEM163*, Alzheimer's disease, rs6430538, genome-wide association study, expression quantitative trait loci, multi-omics data

## Abstract

**Introduction:**

Growing evidence highlights a potential genetic overlap between Alzheimer's disease (AD) and Parkinson's disease (PD); however, the role of the PD risk variant rs6430538 in AD remains unclear.

**Methods:**

In Stage 1, we investigated the risk associated with the rs6430538 C allele in seven large-scale AD genome-wide association study (GWAS) cohorts. In Stage 2, we performed expression quantitative trait loci (eQTL) analysis to calculate the cis-regulated effect of rs6430538 on *TMEM163* in both AD and neuropathologically normal samples. Stage 3 involved evaluating the differential expression of *TMEM163* in 4 brain tissues from AD cases and controls. Finally, in Stage 4, we conducted a transcriptome-wide association study (TWAS) to identify any association between *TMEM163* expression and AD.

**Results:**

The results showed that genetic variant rs6430538 C allele might increase the risk of AD. eQTL analysis revealed that rs6430538 up-regulated *TMEM163* expression in AD brain tissue, but down-regulated its expression in normal samples. Interestingly, *TMEM163* showed differential expression in entorhinal cortex (EC) and temporal cortex (TCX). Furthermore, the TWAS analysis indicated strong associations between *TMEM163* and AD in various tissues.

**Discussion:**

In summary, our findings suggest that rs6430538 may influence AD by regulating *TMEM163* expression. These discoveries may open up new opportunities for therapeutic strategies targeting AD.

## 1 Background

Potential genetic association may exist between common neurodegenerative diseases (Small and Petsko, 2015; Brainstorm et al., 2018; Guo et al., 2022). Alzheimer's disease (AD) and Parkinson's disease (PD) are both classic age-related neurodegenerative diseases (Scheltens et al., 2016; Poewe et al., 2017; Hu et al., 2021; Qiu et al., 2022a). Although the pathogenesis of AD and PD differs, they share certain similar pathways in the development of neurodegeneration (Xie et al., 2014). One of the most important pathological features of AD is the deposition of amyloid-β (Aβ), which is also present in the brains of PD patients (Compta et al., 2011; Yu et al., 2014). Similarly, the pathological features of PD are also observed in cases of AD (Lippa et al., 1997). Currently, the shared susceptibility loci of AD and PD, such as *APOE* and *MAPT*, have been identified, indicating a possible overlap in genetic background and pathological characteristics (Laws et al., 2007; Zhu et al., 2017). However, further exploration of additional genetic links is necessary.

Members of the transmembrane (*TMEM*) protein family have been identified as being associated with various neurodegenerative disorders (Chang et al., 2017; Nalls et al., 2019; Li et al., 2020). Among these genes, *TMEM163* serves as an important susceptibility locus for PD. It plays a regulatory role in ATP-evoked behavior in neurons, as well as binding to Zn^2+^ and recruiting them to vesicular organelles (Chang et al., 2017; Salm et al., 2020; Kia et al., 2021; Mammadova-Bach and Braun, 2021). Notably, brain zinc has been found to be involved in the pathogenesis of AD by influencing amyloid metabolism (Sensi et al., 2018; Xu et al., 2019). It is plausible to hypothesize that rs6430538, through its impact on the expression level of zinc transporters, contributes to the aggravation of Aβ deposition and toxicity by regulating nearby genes. In an attempt to identify shared pathogenic genetic variants between PD and AD, Zhu et al. replicated the association of rs6430538 (*ACMSD-TMEM163*) with AD in a population study (Zhu et al., 2017). They investigated a sample comprising 992 sporadic AD patients and 1,358 controls from northern China and observed a protective effect of rs6430538 on AD (OR = 0.340, *P* = 0.015) (Zhu et al., 2017). Unfortunately, upon regression adjustment for confounding factors including age, gender, and *APOE* ε4 status, rs6430538 did not achieve statistical significance (*P* = 0.072) (Zhu et al., 2017). It is important to note, however, that the sample size of the independent case-control study was relatively small compared to genome-wide association studies (GWAS). Furthermore, previous investigations solely focused on exploring the potential risk association between rs6430538 and AD, neglecting the possibility that rs6430538 may influence AD through the regulation of gene expression.

In this study, we aimed to delve deeper into the association between the PD risk variant rs6430538 and AD. Specifically, we sought to investigate the impact of rs6430538 on AD by analyzing its influence on gene expression using comprehensive datasets, including large-scale GWAS, expression quantitative trait loci (eQTL), and RNA-seq datasets. By doing so, our findings would contribute to a better understanding of the functional role of non-coding region variants, such as rs6430538, and their implications for disease regulation.

## 2 Materials and methods

### 2.1 AD GWAS datasets

We investigated the risk of rs6430538 in seven large-scale GWAS for AD, including two GWAS meta-analyses by International Genomics of Alzheimer's Project (IGAP), three genome-wide association studies by proxy (GWAX) by UK Biobank, and two meta-analyses of GWAS and GWAX (Lambert et al., 2013; Marioni et al., 2018; Buniello et al., 2019; Jansen et al., 2019; Kunkle et al., 2019; Schwartzentruber et al., 2021). UK Biobank used self-reported family history of Alzheimer's disease as the standard of cases, while IGAP used Aβ and tau levels as the standard of cases (the NINCDS-ADRDA criteria or DSM-IV guidelines) (Lambert et al., 2013). Detailed GWAS information was shown in the original studies and Table 1.

**Table 1:** Association between rs6430538 and AD in different GWAS studies.

**Datasets**	**Traits**	**Consortium**	**Cases**	**Controls**	**Beta**	**SE**	***P* value**
IGAP2013	GWAS	IGAP	25,580	48,466	0.0320	0.0088	2.80E-04
IGAP2019	GWAS	IGAP	35,274	59,163	0.0503	0.015	5.82E-04
Jansen et al.	GWAS+GWAX	IGAP 2013, PGC-ALZ, ADSP, and UK Biobank	71,880	383,378	0.00767	0.0021	3.79E-04
Schwartzentruber et al.	GWAS+GWAX	IGAP 2019 and UK Biobank	75,024	397,844	0.0423	0.0097	1.43E-05
UK Biobank (family history)	GWAX	UK Biobank	42,034	272,244	0.0265	0.010	1.04E-02
UK Biobank (maternal history)	GWAX	UK Biobank	27,696	260,980	0.0268	0.013	3.64E-02
UK Biobank (paternal history)	GWAX	UK Biobank	14,338	245,941	0.0256	0.018	1.39E-01

Beta is the regression coefficient based on the effect allele. Beta > 0 and beta < 0 mean the effect allele could increase and reduce AD risk, respectively. SE, standard error. GWAX, genome-wide association studies by proxy. GWAS, genome-wide association studies. The statistically significant association is defined to be P < 0.05/7 = 7.14E-03.

### 2.2 eQTL datasets

We obtained eQTL datasets of normal brains in 13 normal brain tissues from Genotype-Tissue Expression (GTEx), including amygdala, anterior cingulate cortex, caudate, cerebellar hemisphere, cerebellum, cortex, frontal cortex, hippocampus, hypothalamus, nucleus accumbens, putamen, spinal cord and substantia nigra. The donors were of multiple descents including European (85.3%), African (12.3%), Asian (1.4%), etc. and only about 1.2% of the donors died of neurological diseases (GTEx Consortium, 2017).

We obtained eQTL datasets of AD and non-AD samples from Mayo RNAseq Study (MAYO) and Religious Orders Study and Memory and Aging Project (ROSMAP) (Bennett et al., 2012a,b; Zou et al., 2012; GTEx Consortium, 2017; Ng et al., 2017). Mayo RNAseq Study collected the cerebellar (CER) (197 cases and 177 controls) and temporal cortex (TCX) (202 cases and 197 controls) tissues of AD individuals and non-AD individuals, respectively (Zou et al., 2012). Non-AD subjects contained several brain pathologies, such as frontotemporal dementia (FTD), multiple system atrophy and vascular dementia (Zou et al., 2012). ROSMAP collected samples from the dorsolateral prefrontal cortex (DLPFC) of 494 participants (Ng et al., 2017). 97% of the samples were diagnosed with pathological or clinical AD (Ng et al., 2017). The donors were all of European descent.

### 2.3 RNA expression datasets

RNA-seq datasets for AD and controls were generated from AlzData and gene expression omnibus (GEO) databases, including entorhinal cortex (EC) (GSE26927, GSE26972, GSE48350, and GSE5281), hippocampus (HIP) (GSE28146, GSE29378, GSE36980, GSE48350, and GSE5281), TCX (GSE29652, GSE36980, GSE37263, and GSE5281), and Frontal Cortex (FCTX) (GSE12685, GSE36980, GSE48350, GSE5281, GSE53890, and GSE66333) (Xu et al., 2018; Zhang et al., 2019). All differential expression results were adjusted for age and sex of samples.

### 2.4 Statistical analysis

#### 2.4.1 Genetic association of rs6430538 with AD

We used seven GWAS datasets for AD to evaluate the genetic association between rs6430538 C allele and AD. In addition, we carried out gender stratification analysis using GWAX datasets diagnosed by paternal history and maternal history from UK Biobank (Marioni et al., 2018). The statistically significant association is defined to be *P* < 5E-08 after adjusting for multiple testing.

#### 2.4.2 eQTL analysis of rs6430538 on TMEM163

Disease states are known to alter the expression of specific genes (Gratuze et al., 2018; Liu et al., 2019; Hu et al., 2020; Ma and Qiu, 2022; Qiu et al., 2022b,c). Here, we calculated the regulated effect of rs6430538 C allele on *TMEM163* in neuropathologically normal and neuropathologically disease individuals, respectively, by applying linear regression based on an additive model (Hu et al., 2020; Qiu et al., 2022b). According to the additive model, each allele has an independent effect on the trait. We coded the possible genotypes of rs6430538 (CC = 2, CT = 1, TT = 0), where C is an effect allele and T is a non-effect allele. The statistically significant association is defined to be *P* < 0.05/18 = 2.78E-03.

#### 2.4.3 Gene expression analysis of TMEM163

We evaluated the differential expression of *TMEM163* in four brain tissues between AD cases and controls. All differential expression results were adjusted for sex with an FDR of 0.05.

#### 2.4.4 Transcriptome-wide association study analysis

TWAS is a test for a significant association between cis components of gene expression and traits (Gusev et al., 2016). TWAS integrates GWAS and eQTL datasets, not only mining significant correlations between eQTLs and SNPs, but also capturing complete cis-SNP signals. We looked for evidence of the association between *TMEM163* and AD in TWAS using FUSION software (Gusev et al., 2016). Herein, we integrated AD GWAS with gene expression dataset from GTEx and ROSMAP for TWAS analysis (Gusev et al., 2016; GTEx Consortium, 2017; Kunkle et al., 2019). The statistically significant association is defined to be *P* < 5E-08 after multiple testing.

## 3 Results

### 3.1 Rs6430538 regulated the expression of *TMEM163* and increased AD risk

**Figure 1:**
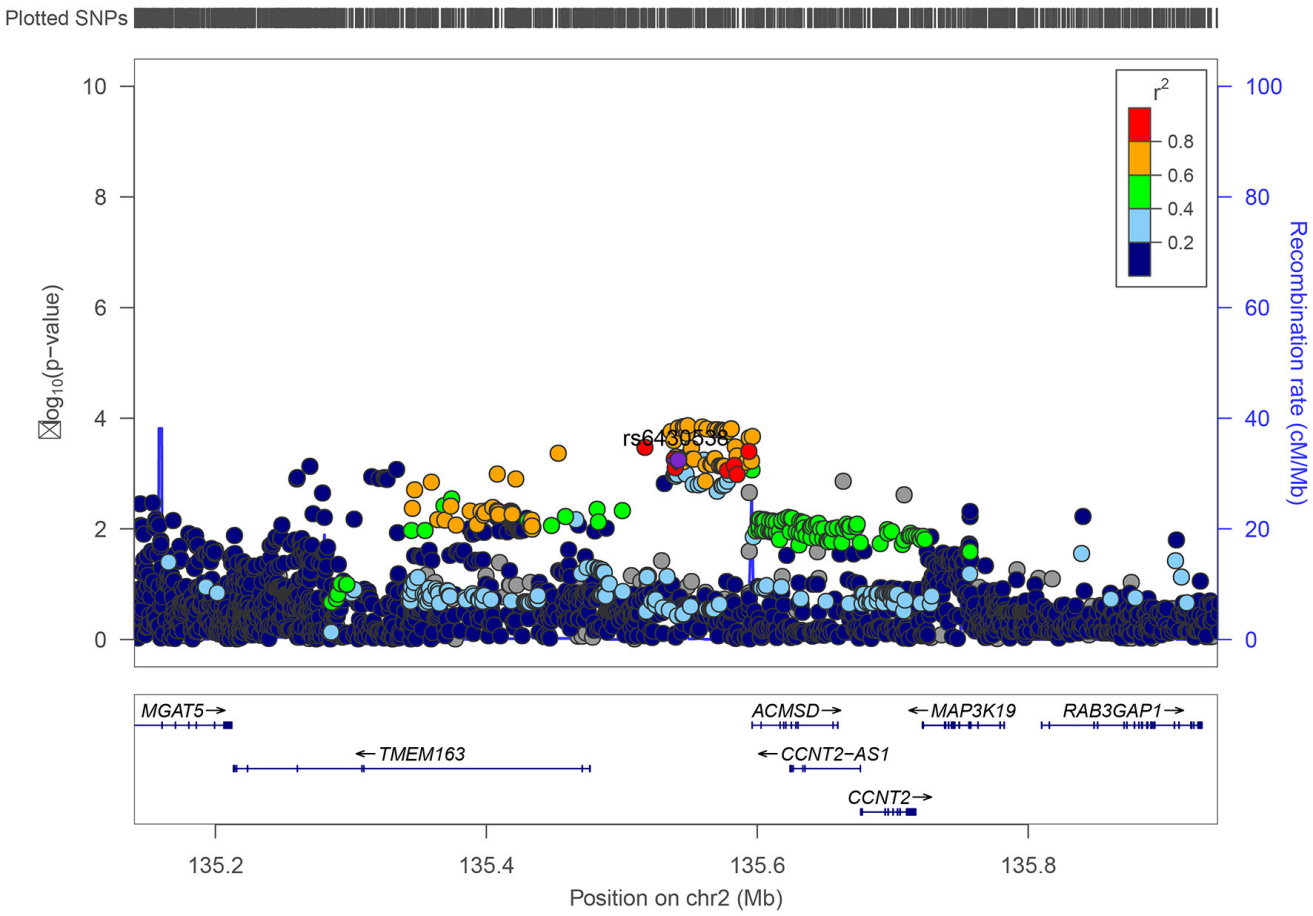
Regional visualization of genome-wide association scan results near variant rs6430538.

We inquired proxy SNPs of rs6430538 and the annotations information on noncoding genome using HaploReg4.1 (Ward and Kellis, 2016). Rs6430538 is located in the intergenic region on chromosome 2, with eight genetic variants highly linked to rs6430538 (r^2^ > 0.8) (Figure 1, Supplementary Table 1) (Pruim et al., 2010). In addition to the GWAX studies by UK Biobank, rs6430538 C allele was suggestively associated with the risk of AD in four large-scale GWAS meta-analyses (Table 1). Moreover, rs6430538 showed no sex-specific differences in AD risk (Table 1).

Based on all brain eQTL studies, rs6430538 involved in regulating the expression of *TMEM163, CCNT2, VDAC2P4, CCNT2-AS1*. Herein, we focused on *TMEM163*, the shared regulated gene, in all the eQTL studies. The eQTL analysis showed that rs6430538 regulated *TMEM163* overexpression in AD patients (Beta = 0.325, *P* = 1.31E-13), while rs6430538 inhibited *TMEM163* expression in non-AD individuals (Table 2). The opposing effects of the same variant may be explained if there is dysregulation of *TMEM163* in disease conditions.

**Table 2:** The regulatory effects of rs6430538 variant C allele on *TMEM163* in both AD and neuropathologically normal individuals.

**Datasets**	**Beta**	***P* value**	**Brain tissue**	**Number**
Mayo	0.0049	8.14E-02	Cerebellum (AD)	186
	−0.0072	4.52E-03	Cerebellum (non-AD)	170
	0.0039	3.47E-01	Temporal cortex (AD)	191
	0.0043	2.87E-01	Temporal cortex (non-AD)	181
ROSMAP	0.325	1.31E-13	DLPFC (AD)	494
GTEx	−0.27	1.0E-02	Brain—Amygdala (normal)	88
	−0.17	1.4E-02	Brain—Anterior cingulate cortex (normal)	109
	−0.31	3.2E-05	Brain—Caudate (normal)	144
	−0.11	1.4E-02	Brain—Cerebellar Hemisphere (normal)	125
	−0.14	8.1E-03	Brain—Cerebellum (normal)	154
	−0.25	3.9E-06	Brain—Cortex (normal)	136
	−0.16	1.5E-03	Brain—Frontal Cortex (normal)	118
	0.043	4.4E-01	Brain—Hippocampus (normal)	111
	−0.19	2.9E-03	Brain—Hypothalamus (normal)	108
	−0.22	1.4E-03	Brain—Nucleus accumbens (normal)	130
	−0.27	5.8E-04	Brain—Putamen (normal)	111
	0.047	5.9E-01	Brain—Spinal cord (normal)	83
	−0.0023	9.8E-01	Brain—Substantia nigra (normal)	80

Beta is the regression coefficient based on the effect allele. Beta > 0 and beta < 0 mean that this effect allele could increase and reduce gene expression, respectively. The statistically significant association is defined to be P < 0.05/18 = 2.78E-03.

### 3.2 *TMEM163* was associated with AD and differentially expressed in AD and normal individuals

**Figure 2:**
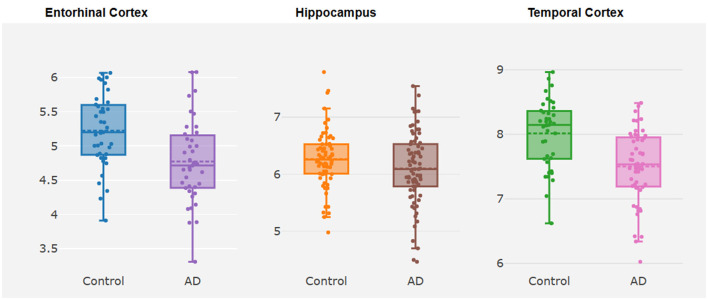
Box plots about differential expression analysis of *TMEM163*. The y-axis represents the expression level of *TMEM163*, while the x-axis represents different brain tissues.

*TMEM163* was differentially expressed in EC ($\log_{2}^{FC}$ = −0.44, *P* = 0.01) and TCX ($\log_{2}^{FC}$ = −0.52, *P* = 4.32E-05) of AD vs. controls (Figure 2, Supplementary Table 2). However, *TMEM163* was not expressed in FCTX. Moreover, we used TWAS to prioritize potential susceptibility genes for AD, and *TMEM163* was suggestively associated with AD in 5 GTEx tissues (whole blood, esophagus muscularis, heart atrial appendage, pituitary and testis) and brain tissues (Table 3). Importantly, *TMEM163* was a potential casual gene of AD in brain (ROSMAP) (Z_TWAS_ = −3.36, *P*_*TWAS*_ = 5.63E-03) and whole blood (Z_TWAS_ = −2.77, *P*_*TWAS*_ = 7.92E-04) (Table 3).

**Table 3:** TWAS analysis results of *TMEM163*.

**Panel**	**Z_eQTL/GWAS_**	**Z_TWAS_**	***P_*TWAS*_***	**HSQ**
GTEx.Brain_Cerebellum	−0.41	−0.20	8.4E-01	0.436
GTEx.Esophagus_Muscularis	2.76	−3.72	1.96E-04	0.088
GTEx.Heart_Atrial_Appendage	2.68	−2.78	5.38E-03	0.313
GTEx.Pituitary	−3.31	−3.44	5.92E-04	0.372
GTEx.Testis	−2.95	3.29	1.01E-03	0.288
GTEx.Whole_Blood	2.76	−3.36	7.92E-04	0.11
ROSMAP.Brain	2.04	−2.77	5.63E-03	0.195

HSQ, Heritability of the gene; Z_eQTL/GWAS_, GWAS Z-score for this eQTL; Z_TWAS_, TWAS Z-score; P_TWAS_, TWAS P-value.

## 4 Discussion

Neurodegenerative diseases, such as AD and PD, share certain genetic associations and pathological characteristics, despite differences in their pathogenesis (Majd et al., 2015; Zhu et al., 2017; Han et al., 2018). The identification of shared susceptibility loci, such as *APOE* and *MAPT*, suggests a potential overlap in genetic background and pathological mechanisms between these diseases. However, further exploration is required to uncover additional genetic links.

Based on the hypothesis that rs6430538, a risk variant associated with PD, may impact AD through the regulation of nearby genes, we aimed to investigate the influence of rs6430538 on AD by analyzing its effect on gene expression using large-scale datasets. To examine the genetic association of rs6430538 with AD, we analyzed seven GWAS datasets, including those from the IGAP and UK Biobank. Our findings indicated that the rs6430538 C allele was associated with an increased risk of AD. Additionally, gender stratification analysis revealed no sex-specific differences in the association between rs6430538 and AD risk. Understanding gender-specific and nongender-specific effects could guide the development of sex-specific therapeutic strategies, taking into account potential differences in disease mechanisms and responses to treatment between males and females.

Although GWAS analysis indicated only suggestive association between rs6430538 and AD, stringent GWAS thresholds might obscure some genetic variants that affect AD through regulating gene expression. Therefore, we investigated the impact of rs6430538 on the expression of *TMEM163* and other nearby genes using eQTL datasets. The results demonstrated that rs6430538 played a role in regulating the expression of *TMEM163, CCNT2, VDAC2P4*, and *CCNT2-AS1*. Specifically, rs6430538 exhibited a regulatory effect on *TMEM163* expression, with overexpression observed in AD patients and inhibited expression in non-AD individuals. As revealed in previous studies, genetic variants linked to AD risk that modulate the expression of genes in human tissues, emphasizing the importance of non-coding regions in disease pathogenesis (Raj et al., 2014). To prioritize potential susceptibility genes for AD, we conducted a TWAS using GWAS and eQTL datasets. Our results indicated that *TMEM163* was strongly associated with AD in multiple tissues, including brain tissues and whole blood.

However, our study had certain limitations. First of all, gender, age (or age of death), disease status (or cause of death) and ancestry, might lead to different gene expression levels in different eQTL datasets. We could not control all the variables. For example, 1.2% of the donors in GTEx died of neurological diseases, and these samples with abnormal neuropathology might have some effects. Similarly, due to the lack of sex stratified eQTLs and original genotype data, it was difficult to deeply explore the contribution of female genotype and gene expression in AD. Furthermore, although the PD susceptibility locus *TMEM163* significantly affected AD in brain tissue and blood tissue, to our knowledge, none of the known methods claimed to find causal gene (Gusev et al., 2016; Wainberg et al., 2019; Zhu et al., 2021). Novel methods of exploring causal genes may be more powerful evidence for our research.

## 5 Conclusions

Genetic variants located in non-coding region may affect traits by regulating gene expression. Here, we comprehensively analyzed the effect of a PD risk variant rs6430538 on AD through regulating gene expression. We demonstrated that different neuropathological samples played different roles in the regulation of gene expression by genetic variants. These findings highlighted the possible genetic overlap between neurodegenerative diseases.

## Data availability statement

The original contributions presented in the study are included in the article/Supplementary material, further inquiries can be directed to the corresponding author.

## Author contributions

SQ: Data curation, Formal analysis, Investigation, Writing – original draft, Writing – review & editing. MS: Conceptualization, Writing – review & editing. YX: Formal analysis, Writing – review & editing. YH: Conceptualization, Funding acquisition, Supervision, Writing – review & editing.
