# Supplementary material for: Integrating multi-omics data to reveal the effect of genetic variant rs6430538 on Alzheimer's disease risk
Source: Front Neurosci. 2024 Mar 18;18:1277187. doi: 10.3389/fnins.2024.1277187 (PMC10982421; doi:10.3389/fnins.2024.1277187)
Supplement: Supplementary file 1 [file Table_1.DOCX]

**Supplementary Table 1.** **Eight variants tagged by** **rs6430538 with r2 ≥ 0.8.**

| **chr** | **pos**  **(hg38)** | **LD**  **(r²)** | **LD**  **(D')** | **variant** | **Ref** | **Alt** | **AFR**  **freq** | **AMR freq** | **ASN**  **freq** | **EUR**  **freq** |
| --- | --- | --- | --- | --- | --- | --- | --- | --- | --- | --- |
| 2 | 134782397 | 1 | 1 | **rs6430538** | C | T | 0.98 | 0.69 | 0.98 | 0.49 |
| 2 | 134779549 | 1 | 1 | rs6741007 | T | G | 0.98 | 0.69 | 0.98 | 0.49 |
| 2 | 134782976 | 1 | 1 | rs7599054 | A | G | 0.98 | 0.69 | 0.98 | 0.49 |
| 2 | 134780328 | 0.98 | 1 | rs6751833 | G | T | 0.94 | 0.64 | 0.71 | 0.48 |
| 2 | 134790951 | 0.96 | 1 | rs146112546 | C | CAAG | 0.95 | 0.67 | 0.96 | 0.48 |
| 2 | 134823942 | 0.96 | 0.98 | rs4953936 | C | T | 0.97 | 0.68 | 0.98 | 0.49 |
| 2 | 134818984 | 0.95 | 0.98 | rs6753334 | A | G | 0.93 | 0.67 | 0.96 | 0.48 |
| 2 | 134834675 | 0.95 | 0.98 | rs6758044 | T | C | 0.96 | 0.68 | 0.97 | 0.49 |
| 2 | 134757922 | 0.83 | 0.95 | rs1942041 | T | C | 0.98 | 0.69 | 0.98 | 0.51 |

**Supplementary Table 2. Differential expression analysis of *TMEM163*.**

| **Brain region** | **log2 Fold Change** | ***P* value** | **FDR** |
| --- | --- | --- | --- |
| **Entorhinal Cortex** | **-0.44** | **1.00E-02** | **0.018** |
| Hippocampus | -0.15 | 1.32E-01 | 0.352 |
| **Temporal Cortex** | **-0.52** | **4.32E-05** | **0.001** |
